# Supplementary material for: Impacts of Dietary Standardized Ileal Digestible Lysine to Net Energy Ratio on Lipid Metabolism in Finishing Pigs Fed High-Wheat Diets
Source: Animals (Basel). 2024 Jun 19;14(12):1824. doi: 10.3390/ani14121824 (PMC11200874; doi:10.3390/ani14121824)
Supplement: Supplementary file 1 [file animals-14-01824-s001.zip › animals-2950952-supplementary.pdf]

animals

MDPI

Supplementary Materials

Table S1. Effects of dietary standardized ileal digestible lysine to net energy ratio on growth performance of finishing pigs.

Items	HR	LR	*P*-value
65-90 kg			
ADG, kg [1]	1.13±0.05	1.11±0.04	0.697
ADFI, kg [2]	3.31±0.15	3.11±0.06	0.31
F:G [3]	2.922±0.038	2.820±0.084	0.316
90-110 kg			
ADG, kg	1.09±0.05	1.03±0.03	0.355
ADFI, kg	3.58±0.15	3.21±0.06	0.088
F:G	3.280±0.008	3.113±0.084	0.229
65-110 kg			
ADG, kg	1.11±0.05	1.08±0.02	0.506
ADFI, kg	3.42±0.15	3.15±0.05	0.167
F:G	3.067±0.023	2.931±0.063	0.112

Note: [1] ADG: average daily gain. [2] ADFI: average daily feed intake. [3] F:G: the ratio of feed to gain.

Table S2. Primers used for real-time PCR analyzing.

Genes	Primers	Sequences (5′-3′)	Length (bp)	Accession number
AMPKα1	Forward	TTGACTCGGCCCCATCCT	65	NM_001167633.1
	Reverse	GTATGGCGTGCCCTTGGA		
SIRT1	Forward	GGTTTGAAGAATGTTGCCTG	114	NM_001145750.2
	Reverse	CCGTTTACTAATCTGCTCCT		
PGC-1α	Forward	GCCCAGTCTGCGGCTATTT	265	NM_213963.2
	Reverse	GTTCAGCTCGGCTCGGATTT		
FXR	Forward	TATGAACTCAGGCGAATGCCTGCT	154	NM_001287412.1
	Reverse	ATCCAGATGCTCTGTCTCCGCAAA		
SHP	Forward	TGCTGCCTGGAGTCCTTATG	269	XM_003127720.4
	Reverse	ACAGGGCGAAAGAAGAGGTC		
CYP7A1	Forward	GAAAGAGAGACCACATCTCGG	123	NM_001005352.3
	Reverse	GAATGGTGTTGGCTTGCGAT		
CYP27A1	Forward	ACTGAAGACCGCGATGAAAC	106	NM_001243304.1
	Reverse	CAAAGGCGAATCAGGAAGGG		
CYP8B1	Forward	GCAGGCGGAGGAGTTATTCA	164	NM_214426.1
	Reverse	TTATGCCGTGCCTCTCCAAG		
CYP7B1	Forward	ATGTGAGAAGATGGTTGGGGT	86	XM_021089297.1
	Reverse	CCAATGAAAGGAAGCCAGCTTTT		
PPARα	Forward	CATCCTCGCGGGAAAGG	70	NM_001044526.1
	Reverse	GGCCATACACAGTGTCTCCAT		
FABP4	Forward	TGGAAACTTGTCTCCAGTG	147	NM_001002817.1
	Reverse	GGTACTTTCTGATCTAATGGTG		
CD36	Forward	AACTACCTTCCTTAGACCTCAGA	117	NM_001044622.1
	Reverse	CAATGACAGCACCAGCAATG		
SCD	Forward	TTCCCCAAAGCCTGTTCGTC	170	NM_213781.1
	Reverse	GTGGAAGCCCTCACCCACAG		
ACCα	Forward	GTTTCCAGACTCTGCGTTTAAG	84	NM_001114269.1

	Reverse	CAGTTGGCAAAGACCATTAGAG		
ACCβ	Forward	GTTTGCAGAGGATCGCATTTAC	247	NM_001206399.1
	Reverse	CTCATTCTGCAGGTACTCGAAG		
FATP2	Forward	GGTCGACTCTTTCCTAGACAAA	84	NM_001278777.1
	Reverse	GTGGAAAAGTTAACTTCCGACC		
HSL	Forward	ACCCTCGGCTGTCAACTTCTT	84	NM_214315.3
	Reverse	TCCTCCTTGGTGCTAATCTCGT		
ATGL	Forward	TCACCAACACCAGCATCCA	95	NM_001098605.1
	Reverse	GCACATCTCTCGAAGCACCA		
CPT	Forward	GCATTTGTCCCATCTTTCGT	198	NM_001129805.1
	Reverse	GCACTGGTCCTTCTGGGATA		
GAPDH	Forward	GTCGGAGTGAACGGATTTGGC	150	NM_001206359.1
	Reverse	CTTGCCGTGGGTGGAATCAT		

Note: AMPKα1: AMP-activated protein kinase α1; SIRT1: sirtuin 1; PGC-1α: peroxisome proliferator-activated receptor-γ coactivator-1α; FXR: farnesol X receptor; SHP: small heterodimer partner; CYP7A1: cytochrome P450 family 7 subfamily A member 1; CYP27A1: cytochrome P450 family 27 subfamily A member 1; CYP8B1: cytochrome P450 family 8 subfamily B member 1; CYP7B1: cytochrome P450 family 7 subfamily B member 1; PPARα: peroxisome proliferator-activated receptor family α; FABP4: fatty acid transport protein 4; CD36: fatty acid translocase; SCD: stearoyl-CoA desaturase; ACCα: acetyl-coA carboxylase α; ACCβ: acetyl-coA carboxylase β; FATP2: fatty acid transport protein 2; HSL: hormone-sensitive lipase; ATGL: adipose triglyceride lipase; CPT: carnitine palmitoyl transferase; GAPDH: glyceraldehyde-3-phosphate dehydrogenase.

Table S3. Effects of dietary standardized ileal digestible lysine to net energy ratio on the coefficient of apparent total tract digestibility of nutrients of finishing pigs.

Items	HR	LR	*P*-value
65-90 kg			
Dry matter (%)	79.92±0.33	79.22±1.29	0.620
Gross energy (%)	81.38±0.42	80.73±1.20	0.648
Crude protein (%)	80.22±1.02	76.57±1.89	0.114
Ether extract (%)	29.29±1.02	42.27±3.19*	0.006
Ash (%)	29.42±0.76	26.28±3.62	0.432
90-110 kg			
Dry matter (%)	78.02±0.33	79.62±1.56	0.297
Gross energy (%)	79.12±0.27	79.69±1.78	0.729
Crude protein (%)	81.30±0.80	80.32±2.31	0.674
Ether extract (%)	31.11±1.37	46.64±5.39	0.059
Ash (%)	23.77±3.45	32.40±0.91	0.067

Note: * indicates significant difference ($P < 0.05$).

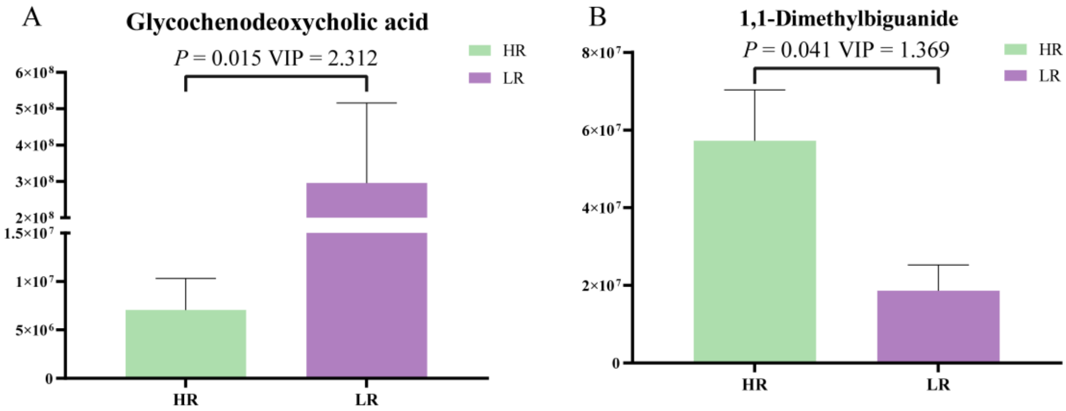

Figure S1. Effects of dietary standardized ileal digestible lysine to net energy ratio on glycochenodeoxycholic acid (A) and 1,1-dimethylbiguanide (B) levels in urine of finishing pigs.
